# Interactive effects of selenium and fertigation regimes on strawberry performance and biofortification under salinity stress

**DOI:** 10.1038/s41598-026-58640-6

**Published:** 2026-07-27

**Authors:** Mohammad Pourebrahimi, Saeid Eshghi, Mohsen Shirdel, Nazim S. Gruda

**Affiliations:** 1 Department of Horticultural Science, School of Agriculture, Shiraz University, P.O. Box 71946- 84334, Shiraz, Iran; 2 Department of Horticultural Sciences, University of Bonn, INRES – Institute of Crop Science and Resource Conservation, 53113 Bonn, Germany

**Keywords:** Superoxide dismutase, Peroxidase, Antioxidant, Anthocyanin, Plant nutrients, Environmental sciences, Physiology, Plant sciences

## Abstract

Salinity is a significant constraint for strawberry production worldwide. This study investigated the effects of selenium (Se) supplementation and different fertigation regimes on strawberry plant performance under saline conditions. The experiment was conducted as a three‑factor factorial arrangement in a completely randomized design with four replicates. Rooted daughter plants of the cv. ‘Paros’ were grown in 3-L pots with a perlite–coir pith mixture and exposed to two salinity levels (control or without NaCl and 40 mM NaCl). Se was supplied as sodium selenate (0 or 1 mg L^− 1^). The third factor was fertigation regime at four levels: morning Hoagland (M-H, 8:00 AM, daily), afternoon Hoagland (A-H, 8:00 PM, daily), both morning and afternoon Hoagland (MA-H, 8:00 AM and 8:00 PM, every other day) and mixed morning/afternoon Hoagland at a 1:2 ratio (MA-1/2H, 8:00 AM and 8:00 PM, daily). Salinity and Se were applied via the nutrient solution. Under salinity, the M-H treatment with Se was associated with reduced yield loss (24%, P < 0.05) and higher root and shoot dry weights compared to other fertigation regimes. This treatment also showed associations with enhanced antioxidant responses, including free radical inhibition (80%), higher anthocyanin content, and increased superoxide dismutase activity, along with improved K and Ca accumulation in shoots. Se supplementation increased fruit Se concentrations to 2.2–3.1 mg kg^− 1^ dry weight. These results indicate that combining Se application with M-H delivery is a promising strategy to mitigate salinity-induced stress in controlled environment systems. Future research is required to evaluate these regime-dependent responses in other cultivars and to optimize Se doses for biofortification while maintaining peak plant performance.

## Introduction

Stress in plants can be described as any abiotic factor or biological limitation that impedes photosynthesis, thereby reducing the plant’s capacity to convert energy into biomass. Given that salinity impacts more than 10% of the global land surface, salt stress is a significant challenge to crop yield and plant productivity^1^. The FAO reports that soil salinization makes roughly 1.5 million ha of agricultural land unusable annually^2,3^. High salt concentrations accelerate senescence and abscission in older leaves, thereby diminishing the photosynthetic source capacity required to maintain growth and development in younger leaves^4^. This reduction ultimately results in decreased branch growth, reduced formation of side branches, and, consequently, lower yield in the strawberry^5^. Treatment with 40 mM NaCl decreased the biomass, yield, and leaf area in the strawberry^6^. Recent studies show that salinity severely disrupts key physiological traits in horticultural crops. In watermelon, salt stress reduces root growth, leaf number, chlorophyll content, and total biomass, accompanied by increased oxidative damage^7^. Similar declines in leaf and root biomass, SPAD values, and relative water content have been reported in cucumber, indicating that salinity adversely affects photosynthesis, water relations, and overall plant performance^8^. In Iran, approximately 6.8 million ha of agricultural land are affected by salinity, of which 4.3 million ha are limited mainly due to salt-related soil problems. Salinity in irrigation water, together with factors such as erosion, unfavorable soil properties, and shallow groundwater levels, further reduces agricultural productivity^9^. As Iran is the second-largest strawberry producer in the Middle East after Turkey, with an annual production exceeding 65,000 tons, understanding the impact of salinity stress on this crop is particularly important^10^.

Various methods have been developed to improve salinity tolerance in crops, including the application of silicon, melatonin, and selenium (Se)^11–13^. While the composition and concentration of different nutrient elements have been extensively investigated to enhance salinity tolerance, different fertigation regimes have received considerably less attention. Fertigation regimes are physiologically important because plant water and nutrient uptake follow circadian rhythms that influence absorption efficiency. Within this framework, the capacity of certain crops to store Se is particularly significant for human health and diet^14^. Strawberries (*Fragaria × ananassa* Duch.), a popular fruit, can store substantial amounts of Se, with concentrations that vary from 0 to 12 µg kg^-1^ of fresh weight^15^. The high nutritional value of strawberries, combined with increased consumer awareness of their health benefits, has led to a rise in per capita strawberry consumption worldwide^16^. The ‘Paros’ strawberry cultivar is recognized as a prominent variety cultivated in Iran, distinguished by large fruit size and high yield potential. Its notable adaptability to off-season production in the southern regions of the country, coupled with its high productivity, has positioned ‘Paros’ as a significant cultivar for research endeavors in strawberry cultivation^17^. One of the major issues in strawberry cultivation is salt stress, as strawberries are sensitive to sodium chloride. Salinity resulting from NaCl negatively impacts plant development and fruit yield^18^.

The application of Se in low concentrations has reportedly improved food storage and development traits in spinach and ryegrass under controlled conditions. This indicates that Se is not just beneficial for stress adaptation but also contributes an important part to promoting plant growth and development^19–22^. Chemically, Se is identical to sulfur, which may influence cellular substitution processes, allowing Se to replace sulfur in sulfur-containing proteins and other chemicals^23^. Research by Zhang HaiYing et al.^24^ demonstrated that strawberries exhibit the highest Se absorption capacity during leaf opening and full flowering stages. Also, Se helps maintain cell membrane integrity by reducing malondialdehyde (MDA) levels, which accumulate due to lipid peroxidation. Moreover, Se can decrease the harmful effects of heavy metals, effectively controlling the accumulation of lead (Pb) and cadmium (Cd) in strawberry fruit and leaves.

The recent concentration of Se in agricultural goods has received major interest due to its critical role in the food chain^15^. Se is a vital element for humans, necessary for the proper functioning of living organisms^25^. The concentrations of Se in mammalian tissues vary from 2.5 mg kg^-1^ in muscles to 0.7 mg kg^-1^ in heart tissue. The adult requires about 50–70 µg of Se per day^26^. A Se-deficient diet may be linked to diseases caused by oxidative injury, such as anemia, impaired immunological function, cardiovascular diseases, diminished fertility in men, and a greater cancer risk. Since Se cannot be directly added to food^27^, enhancing the Se content in plants through various methods is essential. These methods include adding Se to the soil, soaking seeds in Se solutions before cultivation, hydroponic and aeroponic cultures using nutrient solutions containing Se compounds, and foliar application of Se-containing solutions^28^.

Plant nutrition is a critical factor influencing strawberry growth and fruit production. Proper fertilization can lead to higher yields, larger fruit size, and improved fruit quality. This is especially important for potted and hydroponic crops, where nutrient management is more sensitive. Selecting appropriate ratios of elements in the nutrient solution is crucial, as these elements play essential roles in the plant’s biochemical processes, directly and indirectly enhancing yield and product quality^29^. Additionally, irrigation planning and nutrient solution management have a major effect on water use efficiency, productivity, and fruit quality. In targeted production, optimizing irrigation and nutrient management can also serve as a strategy to maximize economic efficiency while improving water productivity^30^. Numerous processes involved in nutrient uptake, transport, and homeostasis are under circadian regulation. Activities such as transpiration, stomatal opening and closure, aquaporin activity, photosynthesis, and the expression of ion transporters exhibit rhythmic patterns that fluctuate throughout the day. These endogenous rhythms cause the plant’s capacity for nutrient uptake and translocation to vary across different times of the day, making nutrient acquisition efficiency inherently time‑of‑day dependent. Accordingly, synchronizing fertigation events with periods of peak metabolic demand and heightened activity of nutrient uptake and transport systems can enhance plant growth, improve nutrient use efficiency, and ultimately increase crop performance^31^. This concept becomes particularly relevant in protected cultivation systems such as greenhouses, where stable environmental conditions allow the circadian regulation of nutrient absorption and associated physiological responses to be observed and evaluated more precisely.

Although the individual effects of Se supplementation and salinity stress on strawberry physiology have been previously explored, the specific role of different fertigation regimes in modulating plant responses to Se under saline conditions remains poorly understood. In particular, no systematic evidence exists on how the scheduling of nutrient solution delivery influences Se accumulation patterns and fruit biofortification. This lack of mechanistic understanding represents a critical research gap that limits the optimization of Se use to improve stress resilience and nutritional quality in strawberries.

We hypothesized that different fertigation regimes under saline conditions influence key physiological responses, particularly antioxidant defense and Se uptake efficiency, thereby enhancing Se biofortification in strawberry fruits. Therefore, this study aimed to elucidate how different fertigation regimes influence physiological responses and Se accumulation in strawberry plants grown under salinity stress, with the broader goal of identifying strategies to improve Se biofortification and plant resilience in salt‑affected production systems.

## Materials and methods

### Research location and experimental design

The study was conducted in a greenhouse in Sirjan County, Iran (29°36′N, 55°50′E, 1780 m). The experimental period was from November 2019 to April 2020. Day and nighttime temperatures in the greenhouse were 23 ± 3/16 ± 3 °C, the relative humidity was 55 ± 5%, and sunlight intensity was 1000 µmol m⁻² s⁻¹. The experiment was conducted as a three-factor factorial with four replications, each consisting of two pots, and each replication was considered an experimental unit. In this experiment, each replication (*n* = 4) consisted of two pots, treated as a single biological replicate (experimental unit). The two pots within each replicate were assigned to different measurement categories to avoid repeated destructive sampling on the same plant. One pot per replicate was designated for leaf-related and biochemical measurements (leaf area, physiological traits, and EDX elemental analysis). In contrast, the second pot was used for destructive biomass measurements (fresh and dry weight of shoots and roots, and fruit quality parameters) and total yield assessment. Subsamples taken from leaves, fruits, or tissues within a pot were considered technical subsamples and were not treated as independent replicates in the statistical analysis. The first factor was salinity at two levels: control (without NaCl) and 40 mM NaCl. Selenium (Se), supplied to the roots via a nutrient solution at two concentrations (0 and 1 mg L^− 1^ sodium selenate (Na_2_SeO_4_)), was the second factor. The third factor was fertigation scheduling regimes, applied at four levels: morning Hoagland solution (M-H), afternoon Hoagland solution (A-H), both morning and afternoon Hoagland solution (MA-H), and a mixed morning/afternoon Hoagland solution at a 1:2 ratio (MA-1/2H). Fertigation treatments were applied at fixed times: 8:00 AM for the M-H and 8:00 PM for the A-H. All treatments, except for the combined MA-H treatment, were applied daily. The MA-H treatment was administered every other day. To ensure comparability among fertigation treatments, the total daily nutrient input was standardized. For the M-H and A-H treatments, plants received V mL of full-strength Hoagland solution once per day. In the MA-H treatment (applied every other day), the total nutrient input over a two‑day cycle was matched by supplying 2 V mL of full-strength solution on fertigation days, resulting in an equivalent average daily nutrient input. For the MA-1/2H treatment, plants received two applications of half-strength Hoagland solution per day (each equivalent to V/2 mL of full-strength), yielding a total daily nutrient input of V mL. Thus, all treatments received the same total amount of nutrients, independent of the fertigation regime. Treatments began when the plants had developed four to five healthy leaves.

### Plant materials

**Fig. 1:**
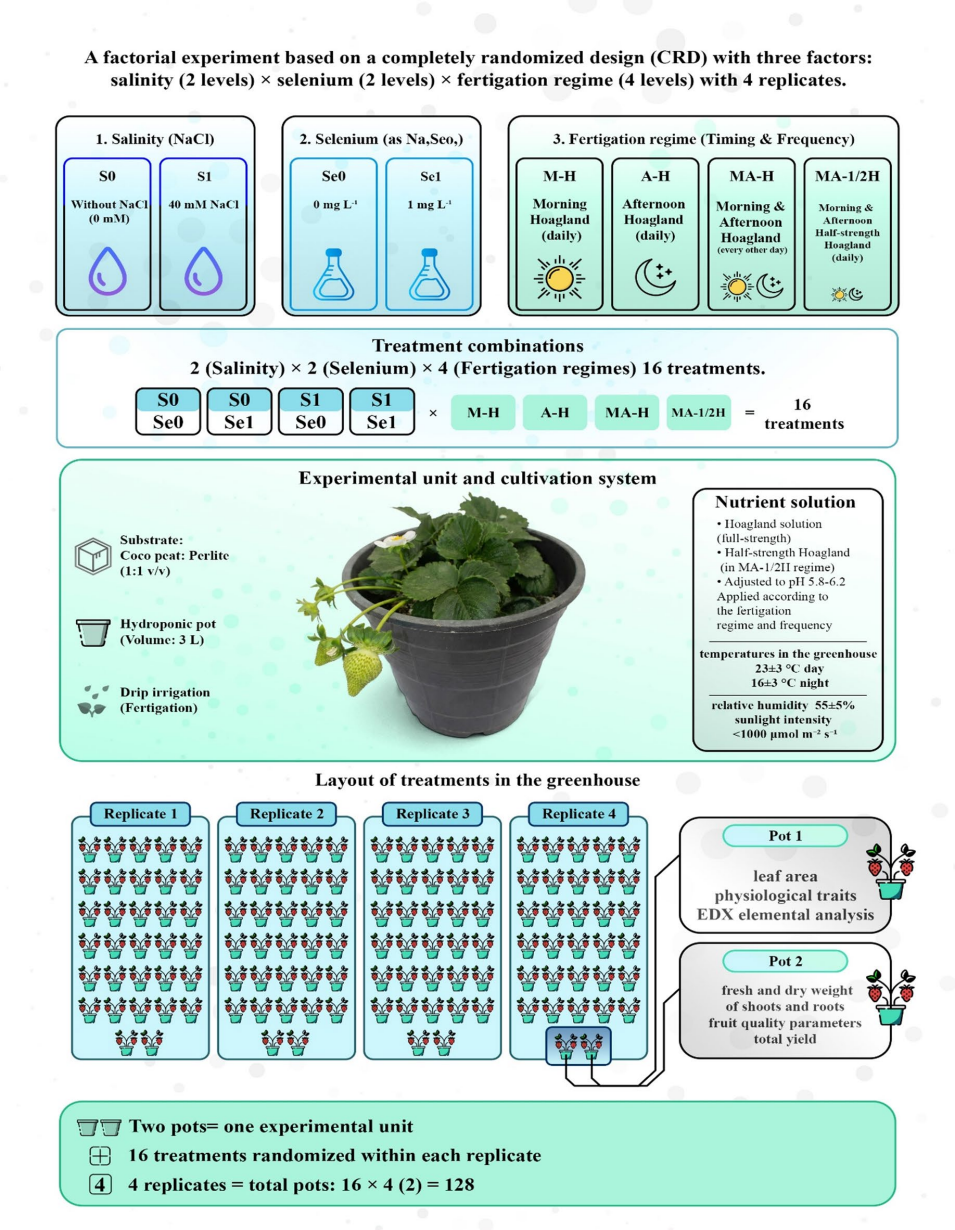
A schematic diagram of the experimental design including salinity (0 and 40 mM NaCl), selenium (0 and 1 mg L^-1^ sodium selenate), and four fertilization regimes (M-H, A-H, MA-H, and MA-1/2H) in 4 replications (two plants each replication), along with plant cultivation, application of treatments, and evaluation of growth, physiological, biochemical, mineral, yield, and selenium biofortification characteristics in the strawberry cultivar ‘Paros’.

For planting, cocopeat and perlite were mixed (v/v 1:1) and put into 3 L pots, and rooted daughter plants of the ‘Paros’ cultivar were planted in these pots. In the initial week, the plants received daily irrigation with unadulterated water. During the second and third weeks, the pots were administered a half-strength Hoagland nutritional solution every other day. From the fourth week onwards, plants were fed fully modified Hoagland nutrients (4 mM Ca(NO₃)₂·4H₂O, 1 mM NH₄H₂PO₄, 6 mM KNO₃, 2 mM MgSO₄·7H₂O, 0.77 µM ZnSO₄·5H₂O, 9.15 µM MnCl₂·4H₂O, 0.50 µM CuSO₄·5H₂O, 0.12 µM H₂MoO₄·H₂O, 46.2 µM H₃BO₃, and 34.2 µM FeEDTA) according to the experimental treatments (Fig. 1) until the study concluded. Plants were irrigated with a nutrient solution at a pH of 6.0 ± 0.2. The quantity of nutrient solution provided was adjusted based on the plant’s developmental stage. Plants in the 2–5 leaf stage received 100 mL, while those at more advanced stages (beyond 5 leaves) were given 150–200 mL. To ensure optimal conditions, 10% of the nutrient solution was drained from the base of the pot after each application. After 40 mM NaCl was added, the EC of the Hoagland solution increased from 1.66 dS/m to 5.21 dS/m. A single irrigation event with water was considered sufficient for leaching after every three nutrient solution applications. Pest and disease control was conducted in accordance with standard local management practices. Flower pollination was carried out manually to ensure uniform fertilization.

### Measured traits

#### Leaf area

Using a leaf area meter (Delta T Devices, UK), five leaves from each plant were gathered, and the leaf area was calculated. The results were presented in cm².

#### Dry weights of roots and shoots

Upon completion of the research, the plants were extracted from their pots, and the roots were meticulously cleansed. After detaching the roots from the shoots, the fresh weight of each was determined individually. For 48 h of heating the samples in an oven set to 70 °C, the dry weights of the roots and shoots were noted.

#### Yield

Mature fruits were picked during approximately 12 weeks (spanning three months) of the fruit development period when 75% of their surface color had become red. The unripe fruits still on the plants at the conclusion of the research period were collected and weighed. The overall yield for each plant during this period was calculated by adding up the total weight of every fruit produced on that plant over the course of the experiment.

#### Plant pigment content

The content of chlorophyll and carotenoids was quantified using a procedure established by Arnon^32^ employing the given equation:

$$\:{\mathrm{Chlorophyll}}\:{\mathrm{a}}\left( {{\mathrm{mg}}\:{\mathrm{g}}^{{{\text{ - 1}}}} \:{\mathrm{FW}}} \right){\text{ = }}\frac{{{\mathrm{12}}{\mathrm{.7}}\left( {{\mathrm{A}}_{{{\mathrm{663}}}} } \right){\text{ - }}\:{\mathrm{2}}.{\mathrm{69}}\:\left( {{\mathrm{A}}_{{{\mathrm{645}}}} } \right) \times {\mathrm{Volume}}\:{\mathrm{made}}}}{{{\mathrm{Wt}}{\mathrm{.}}\:{\mathrm{of}}\:{\mathrm{the}}\:{\mathrm{sample}}\: \times {\mathrm{10}}}}$$

$$\:{\mathrm{Chlorophyll}}\:{\mathrm{b}}\:({\mathrm{mg}}\:{\mathrm{g}}^{{{\text{ - 1}}}} \:{\mathrm{FW}}){\text{ = }}\:\frac{{{\mathrm{22}}{\mathrm{.9}}\:\left( {{\mathrm{A}}_{{{\mathrm{645}}}} } \right){\text{ - 4}}.{\mathrm{68}}\:({\mathrm{A}}_{{{\mathrm{663}}}} \:)\: \times {\mathrm{Volume}}\:{\mathrm{made}}}}{{{\mathrm{Wt}}{\mathrm{.of}}\:{\mathrm{the}}\:{\mathrm{sample}}\: \times {\mathrm{10}}\:}}$$

$$\:{\mathrm{Total}}\:{\mathrm{chlorophyll}}\:({\mathrm{mg}}\:{\mathrm{g}}^{{{\text{ - 1}}}} \:{\mathrm{FW}})\:{\text{ = }}\:\frac{{{\mathrm{20}}{\mathrm{.2}}\:\left( {{\mathrm{A}}_{{{\mathrm{645}}}} } \right){\text{ + 8}}.{\mathrm{02}}\:({\mathrm{A}}_{{{\mathrm{663}}}} \:) \times {\mathrm{Volume}}\:{\mathrm{made}}}}{{{\mathrm{Wt}}{\mathrm{.of}}\:{\mathrm{the}}\:{\mathrm{sample}}\: \times {\mathrm{10}}\:}}$$

$$\:{\mathrm{Carotenoid}}\:({\mathrm{mg}}\:{\mathrm{g}}^{{{\text{ - 1}}}} \:{\mathrm{FW}}){\text{ = }}\:\frac{{{\mathrm{1000}}\left( {{\mathrm{A}}_{{{\mathrm{470}}}} } \right){\text{ - 1}}{\mathrm{.82}}\:{\mathrm{C}}_{{\mathrm{a}}} {\text{ - }}\:{\mathrm{85}}.{\mathrm{02}}\:{\mathrm{C}}_{{\mathrm{b}}} }}{{{\mathrm{198}}}}$$

Dimethyl sulphoxide was used in the dark to extract carotenoids and total chlorophyll. An Epoch Microplate reader (Biotech, USA) was used to measure the leaf extract’s absorbance at 663 nm, 645 nm, and 470 nm.

### Assay antioxidant enzymes peroxidase (POD) and superoxide dismutase (SOD)

One g of leaf tissue, kept at -80 °C, was weighed and homogenized with two mL of 50 mM potassium phosphate buffer (pH 7). The potassium phosphate buffer comprised 2 mM ethylenediaminetetraacetic acid (Na-EDTA) and 1% polyvinylpyrrolidone (PVP). The obtained blend was centrifuged for ten minutes at 4 °C to determine the antioxidant activity of the enzyme, and the supernatant then served as an extract^33^.

The oxidation rate of guaiacol by hydrogen peroxide (H₂O₂) over 2 min at 470 nm was used to calculate POD activity. The reaction mixture used to measure this enzyme included enzyme extract (50 µL), potassium phosphate buffer (2.9 mL, 10 mM), and guaiacol (0.05 mL, 20 mM). The process began with the addition of hydrogen peroxide (20 µL of 40 mM). The expression of POD activity was mM g⁻¹ FW ^33^.

The function of SOD was assessed using 3 mL of a reaction mixture including 250 µL of enzyme extract, 50 mM potassium phosphate, 0.1 mM EDTA, 13 mM L-methionine, 75 µM nitroblue tetrazolium (NBT), and 4 µM riboflavin. Riboflavin was the final material included in the reaction mixture. After the cuvettes were exposed to light (two 15 W fluorescent lamps) for 15 min, the reaction started. Finally, the cuvettes were wrapped using a black cloth. A cuvette, containing the entire but uncoated reaction mixture, was regarded as a blank, while the other cuvette, which had no enzyme extract, was regarded as a control. A spectrophotometer (Biochrom Ltd, Cambridge, UK, Biowave II UV/vis) was used to measure the absorbance of each sample at 560 nm. One unit of enzyme activity is defined as the quantity that reduces the absorbance by 50% relative to the control sample’s absorbance. Unit g^− 1^ FW was used to report the enzyme activity^34^.

### Malondialdehyde concentration (MDA)

After fully powdering 250 mg of the fresh leaf material with liquid nitrogen, five mL of 1% trichloroacetic acid (TCA) was added. At 10,000 rpm for five minutes, the extracted material was centrifuged. Subsequently, 100 µL of the supernatant extract obtained by centrifugation was separated and combined with 400 µL of the MDA mixture. MDA mixture included TBA (7 g) and TCA (0.175 g). The reagent was prepared to a final volume of 35 mL using distilled water. Subsequently, it was immersed in a hot water bath for 30 min. The absorbance values of the samples were measured using a spectrophotometer at wavelengths of 532 and 600 nm, with the MDA content reported in nmol g⁻¹ FW ^35^.

### Anthocyanin content

The pH difference approach was employed to quantify anthocyanin content^36^. Initially, 400 µL of fruit juice was combined with 3600 µL of potassium chloride (0.025 M, pH 1) and sodium acetate (0.4 M, pH 4.5) buffers individually. At 510 and 700 nm, sample absorbance was quantified using an Epoch Microplate reader (Biotech, USA). Using cyanidin-3-glucoside mg in 100 cc of juice from fruit, the anthocyanin amount was calculated.

### Antioxidant activity (%)

Fruit juice’s ability to scavenge free radicals using DPPH (2,2-diphenyl-1-picrylhydrazyl) was used to measure its antioxidant activity. 200 µL of a 0.1 mM DPPH solution was mixed with 22 µL of juice from the fruit extract. Upon the addition of DPPH, the mixture was promptly mixed. The solution had to settle at 25 °C in the dark for half an hour. The T60 UV-visible spectrophotometer (Company Biotech, USA) was used to measure the absorbance at 517 nm. By measuring the % of DPPH inhibition, the extracts’ antioxidant capacity was assessed^37^.

### Mineral nutrient contents

Quantitative determination of mineral nutrients (calcium (Ca), iron (Fe), potassium (K), sodium (Na)) and Se in plant tissues, including shoots, roots, and fruits, was performed using Energy‑dispersive X‑ray Spectroscopy (EDX) integrated with a Scanning Electron Microscope (SEM) (TESCAN Vega‑3, TESCAN, Czech Republic). Plant tissues were harvested, washed, oven‑dried at 70 °C for 48 h to a constant dry weight, and then pulverized to a fine powder. A 1.0 g aliquot of each dried, powdered sample was mounted on an aluminum stub and sputter‑coated with a 10 nm silver layer for conductivity. Analyses were conducted at an accelerating voltage of 20 kV, a beam current of 10 nA, and a working distance of 10 mm, with EDX spectral acquisition lasting 60 s at three randomly selected points and one mapped area per replicate, all performed at the Central Laboratory of Shiraz University. Quantitative analysis relied on validated external calibration using Certified Reference Materials (CRMs) NIST 1573a (Tomato Leaves) and NIST 1575a (Pine Needles) for Ca, Fe, K, Na, and Se, generating calibration curves from peak counts per second (cps) versus certified concentrations (mg kg⁻¹). Raw EDX spectra were processed using the Aztec software package, applying background subtraction and peak deconvolution. For major elements including Na, Ca, and K, atomic percentages (At.%) were converted to mass concentrations expressed as weight% (wt%) of dry weight via calibration curves and ZAF corrections for matrix effects, while Se was reported in mg kg⁻¹ dry weight, ensuring its limit of detection (LOD) was below 0.1 mg kg⁻¹. Validation included instrumental repeatability (RSD < 5% for major elements and < 10% for Fe and Se), recovery studies (92–105%), and routine analysis of CRMs and procedural blanks. Fruit tissue analysis followed the same protocol, with specific attention to selenium calibration and detection within validated ranges. Mean values were computed from four replicates for each sample after analysis^38–40^. Although SEM‑EDX is not the conventional reference technique for absolute quantitative mineral analysis and may have lower sensitivity than ICP‑OES or AAS, its use in this study was determined by instrument availability and budget constraints associated with the large number of samples. The quantitative procedure was strengthened through external calibration with CRMs, ZAF matrix correction, and recovery validation to ensure reliable comparative measurements. Here, we employed SEM‑EDX mainly to assess relative differences in how elements were distributed among the different treatments. Accordingly, the findings are meant for comparison rather than as exact quantifications of absolute elemental concentrations.

### Statistical analyses

Throughout the experiment, environmental conditions, including light, humidity, and temperature, were continuously monitored to ensure uniformity within the greenhouse. To minimize spatial heterogeneity, pots were arranged in a completely randomized design and repositioned every two weeks to mitigate location-specific microclimatic effects. Initially, the data’s normality and homogeneity of variance were examined to ensure the ANOVA assumptions were met. The variance evaluation of the data was performed with SAS software (version 9.1). In the statistical analysis, each replication (consisting of two pots) was treated as a single experimental unit for all factors. Technical subsamples (e.g., multiple leaves per plant for leaf area, multiple fruits for quality analysis, and triplicate EDX scans) were averaged within each biological replicate before analysis and were not considered independent replicates. Thus, the three‑factor factorial ANOVA was performed using four true biological replicates per treatment combination. A three-factor factorial ANOVA was conducted within a completely randomized design with four replicates. The statistical model included the main effects of salt stress (2 levels), selenium application (2 levels), and fertigation regimes (4 levels), along with all two-way and three-way interactions. At a significance level of *P* < 0.05, Duncan’s multiple range test was applied for comparing means (means ± SD). Figures were created using Excel 2021 and GraphPad Prism version 10.4.1 software.

## Results

### Leaf area

**Fig. 2:**
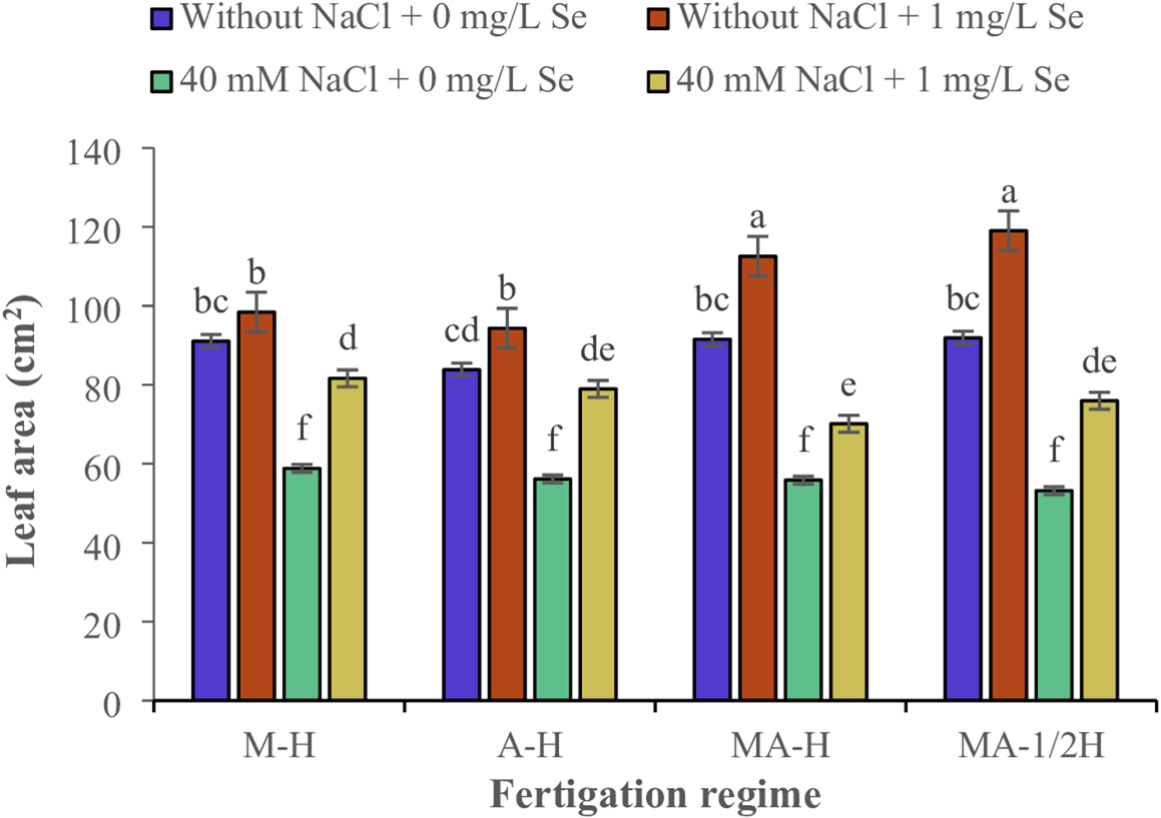
Effect of the three-way interaction of sodium chloride, selenium, and fertigation regime on strawberry leaf area. M‑H: Morning application of Hoagland nutrient solution (daily); A‑H: Afternoon application of Hoagland solution (daily); MA‑H: Morning and afternoon applications of Hoagland solution (every other day); MA‑1/2H: Morning and afternoon applications of half‑strength Hoagland solution (daily). Means (mean ± SD) followed by the same letters are not significantly different according to Duncan’s multiple range test at the 5% significance level (*P* < 0.05).

Salinity, selenium, and fertigation regimes had a significant three-way interaction on leaf area (LA) (*P* < 0.05). Under salinity stress, LA was significantly lower in treatment groups without Se than in Se-supplemented groups. Under salinity stress, the highest LA was observed in the M-H treatment with Se, whereas the MA-1/2H treatment without Se exhibited the lowest value; it was not statistically different from the other fertigation regimes without Se. Under non-saline conditions, the maximum LA was obtained in the MA-1/2H treatment with Se, whereas the smallest amount was found in the A-H treatment without Se (Fig. 2).

### Dry weight of shoots and roots

**Fig. 3:**
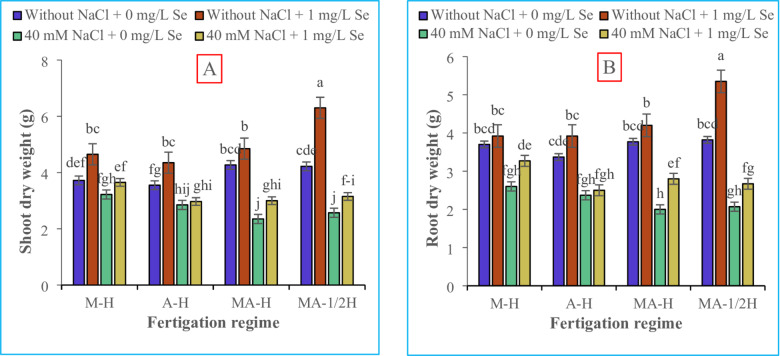
Effect of the three-way interaction of sodium chloride, selenium, and fertigation regime on dry weight of strawberry shoots (A) and roots (B). M‑H: Morning application of Hoagland nutrient solution (daily); A‑H: Afternoon application of Hoagland solution (daily); MA‑H: Morning and afternoon applications of Hoagland solution (every other day); MA‑1/2H: Morning and afternoon applications of half‑strength Hoagland solution (daily). Means (mean ± SD) followed by the same letters are not significantly different according to Duncan’s multiple range test at the 5% significance level (*P* < 0.05).

A significant three-way interaction among salinity, selenium, and fertigation regime was observed for shoot and root dry weight (*P* < 0.05). Treatment with 40 mM sodium chloride markedly decreased the dry weight of roots and shoots. Under salinity stress, the M-H treatment, along with Se, resulted in the highest dry weight values for both roots and shoots (Fig. 3A, B). Conversely, the MA-H treatment without Se had the lowest values under salinity stress. Leaf dry weight in the M-H treatments with Se was 27% higher than in the MA-H treatment without Se under salinity stress. Under non-saline conditions, the minimum and maximum dry weight values were recorded in the A-H treatment without Se and the MA-1/2H treatment with Se, respectively (Fig. 3A, B).

### Yield

**Fig. 4:**
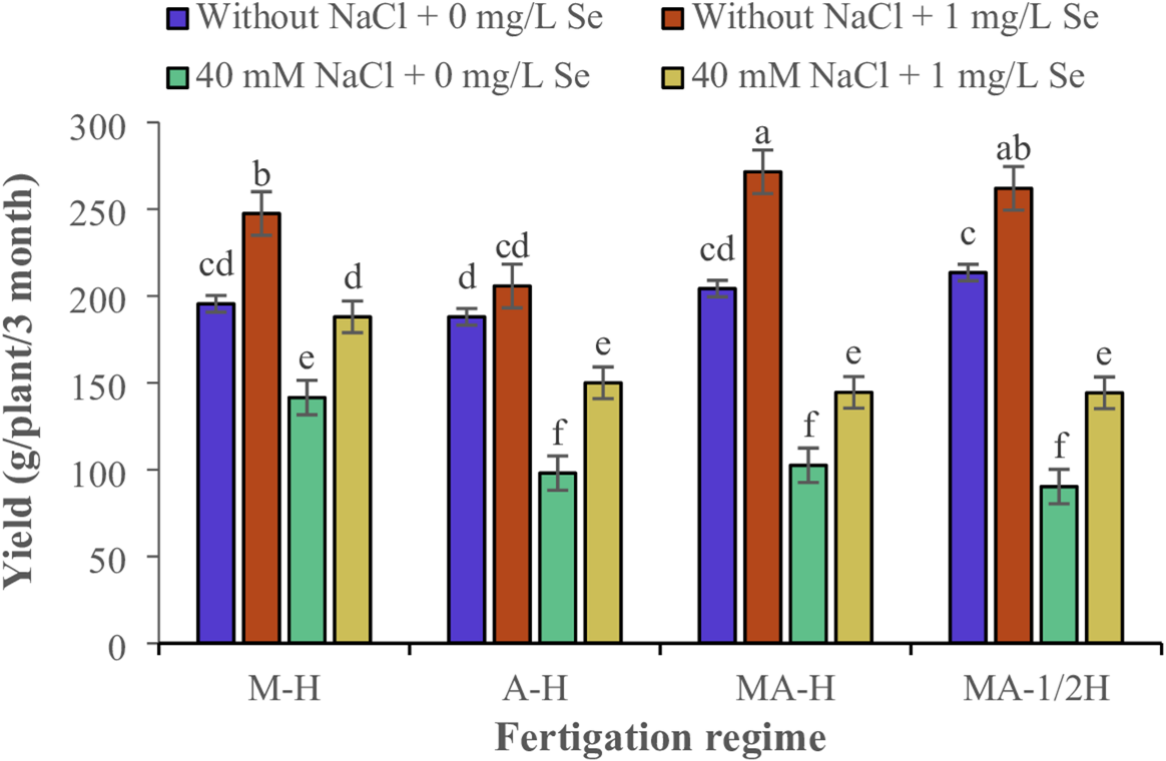
Effect of the three-way interaction of sodium chloride, selenium, and fertigation regime on strawberry yield. M‑H: Morning application of Hoagland nutrient solution (daily); A‑H: Afternoon application of Hoagland solution (daily); MA‑H: Morning and afternoon applications of Hoagland solution (every other day); MA‑1/2H: Morning and afternoon applications of half‑strength Hoagland solution (daily). Means (mean ± SD) followed by the same letters are not significantly different according to Duncan’s multiple range test at the 5% significance level (*P* < 0.05).’

A significant three-way interaction among salinity, selenium, and fertigation regime was observed for yield at the 5% probability level (*P* < 0.05). Salinity stress significantly reduced yield across all treatments. Under non-saline conditions, the MA-H treatment with Se (272 g) achieved the highest yield, which was not significantly different from the MA-1/2H treatment with Se (262 g). Under salinity stress, the M-H treatment with Se recorded the lowest yield reduction (24%) compared to non-saline conditions. In contrast, the MA-1/2H treatment without Se produced the lowest yield and exhibited the highest yield reduction (57%) under salinity stress. Among the Se-free treatments, the M-H treatment produced the highest yield (Fig. 4).

### Plant pigments

A significant three-way interaction among salinity, selenium, and fertigation regime was observed for chlorophyll a (*P* < 0.01), chlorophyll b (*P* < 0.05), total chlorophyll (*P* < 0.05), and carotenoid content (*P* < 0.05). Salinity stress reduced photosynthetic pigment levels across all treatments. The M-H treatment with Se showed slight decreases in chlorophyll a (7%), total chlorophyll (8%), and carotenoids (7%) under salinity stress compared with the corresponding non-saline treatment, although none of these reductions were statistically significant (Table 1). Under non-saline conditions, the MA-1/2H and MA-H treatments with Se showed the highest pigment levels, which were significantly higher than those of the M-H and A-H treatments with Se.

**Table 1:** Effect of the three-way interaction of sodium chloride, selenium, and fertigation regime on chlorophyll *a*, chlorophyll *b*, total chlorophyll, and carotenoid content in strawberry leaves.

**Se (mg L** ^**− 1**^ **)**	**Fertigation regime**	Chlorophyll *a* (mg g^− 1^ F.W.)		Chlorophyll *b* (mg g^− 1^ F.W.)		Total chlorophyll (mg g^− 1^ F.W.)		Carotenoid content (mg g^− 1^ F.W.)	
		**Salinity**							
		Without NaCl	40 mM NaCl	Without NaCl	40 mM NaCl	Without NaCl	40 mM NaCl	Without NaCl	40 mM NaCl
0	M-H	2.05c**	1.49de	0.85abc*	0.42e	2.94b*	1.93c	0.48bc*	0.43cd
	A-H	1.90c	1.25e	0.55de	0.39e	2.48b	1.66c	0.50bc	0.32e
	MA-H	2.00c	1.22e	0.75cd	0.37e	2.79b	1.61c	0.49bc	0.32e
	MA-1/2H	2.05c	1.19e	0.86abc	0.37e	2.95b	1.59c	0.46bc	0.32e
1	M-H	2.10c	1.94c	0.83abc	0.75e	2.97b	2.73b	0.51bc	0.47bc
	A-H	2.07c	1.55d	0.79bcd	0.43e	2.90b	2.01c	0.49bc	0.44c
	MA-H	2.40b	1.48de	1.04ab	0.43e	3.48a	1.94c	0.54b	0.42cd
	MA-1/2H	2.80a	1.41de	1.09a	0.44e	3.93a	1.87c	0.63a	0.35de

*, ** Different letters within each column and row represent significant variations among means using Duncan’s test (*p* ≤ 0.05, *p* ≤ 0.01). M‑H: Morning application of Hoagland nutrient solution (daily); A‑H: Afternoon application of Hoagland solution (daily); MA‑H: Morning and afternoon applications of Hoagland solution (every other day); MA‑1/2H: Morning and afternoon applications of half‑strength Hoagland solution (daily).

### Enzyme activities (SOD, POD) and malondialdehyde (MDA) content

A significant three-way interaction among salinity, selenium, and fertigation regime was observed for SOD activity, POD activity, and MDA content (*P* < 0.05, Table 2). Under salinity stress (40 mM NaCl), the M-H treatment with Se exhibited the smallest increase in MDA content (12.9%), followed by MA-H (40.8%), MA-1/2H (48.8%), and A-H (50.0%) when compared with their corresponding non‑saline treatments. Under the same saline conditions, the M-H treatment with Se also produced the highest SOD and POD activities, consistent with its lower MDA levels. Conversely, under non- saline conditions, the MA-H treatment without Se had the minimum enzyme activities and the maximum MDA content (Table 2).

**Table 2:** Effect of the three-way interaction of sodium chloride, selenium, and fertigation regime on the activities of superoxide dismutase and peroxidase enzymes and malondialdehyde content of strawberry leaves.

Se (mg L^− 1^)	Fertigation regime	SOD (Unit g^− 1^ F.W.)		POD (mM g^− 1^ F.W.)		MAD (nmol. g^−1^FW)	
		Salinity					
		Without NaCl	40 mM NaCl	Without NaCl	40 mM NaCl	Without NaCl	40 mM NaCl
0	M-H	45.85c **	45.40c	33.20fg*	60.46b-e	7.83b-e**	10.7fgh
	A-H	44.85c	46.25c	27.88g	53.88cde	7.9fgh	11.49bc
	MA-H	45.95c	46.10c	49.49def	43.23efg	7.25 g-j	13.93a
	MA-1/2H	45.50c	46.35c	34.14fg	59.52b-e	7.14hij	12.71ab
1	M-H	47.45bc	51.40ab	63.59a-d	79.26a	6.68hij	7.54f-i
	A-H	46.35c	47.87bc	64.85a-d	59.52b-e	6.46hij	9.69de
	MA-H	52.87a	46.55c	65.16a-d	74.56ab	6.17ij	8.69efg
	MA-1/2H	50.60ab	47.60bc	66.41a-d	68.92abc	6.03j	8.97ef

*, ** Different letters within each column and row indicate significant differences among means based on Duncan’s test (*p* ≤ 0.05, *p* ≤ 0.01). M‑H: Morning application of Hoagland nutrient solution (daily); A‑H: Afternoon application of Hoagland solution (daily); MA‑H: Morning and afternoon applications of Hoagland solution (every other day); MA‑1/2H: Morning and afternoon applications of half‑strength Hoagland solution (daily).

### Antioxidant activity and anthocyanin content

**Fig. 5:**
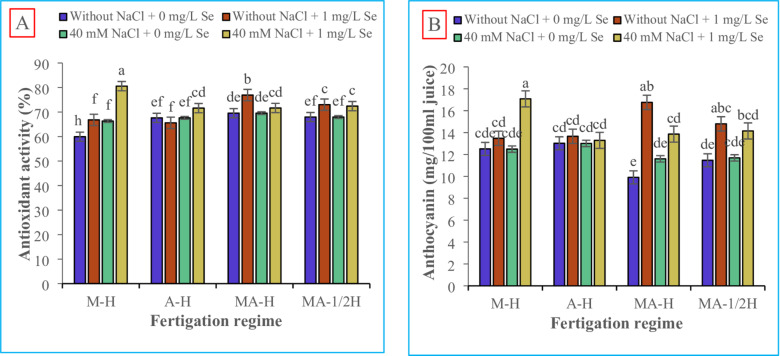
Effect of the three-way interaction of sodium chloride, selenium, and fertigation regime on antioxidant activity (**A**) and anthocyanin content (**B**) in strawberry fruit. M‑H: Morning application of Hoagland nutrient solution (daily); A‑H: Afternoon application of Hoagland solution (daily); MA‑H: Morning and afternoon applications of Hoagland solution (every other day); MA‑1/2H: Morning and afternoon applications of half‑strength Hoagland solution (daily). Means (mean ± SD) followed by the same letters are not significantly different according to Duncan’s multiple range test at the 5% significance level (*P* < 0.05).

The three-way interaction among salinity, selenium, and fertigation regime was significant for antioxidant activity (*P* < 0.01) and anthocyanin content (*P* < 0.05). Under salinity stress, the M-H treatment with Se exhibited a significant increase in antioxidant activity (20%) relative to its corresponding non‑saline treatment. Across Se-treated groups, antioxidant activity was consistently higher than in Se-free treatments under salinity (Fig. 5A). Additionally, the highest anthocyanin content under saline conditions was recorded in the M-H treatment with Se, showing a 21% increase compared with its non-saline control. Under non-saline conditions, the MA-H and MA-1/2H treatments with Se exhibited the highest antioxidant activity and anthocyanin content, respectively (Fig. 5B).

### Sodium concentration

**Fig. 6:**
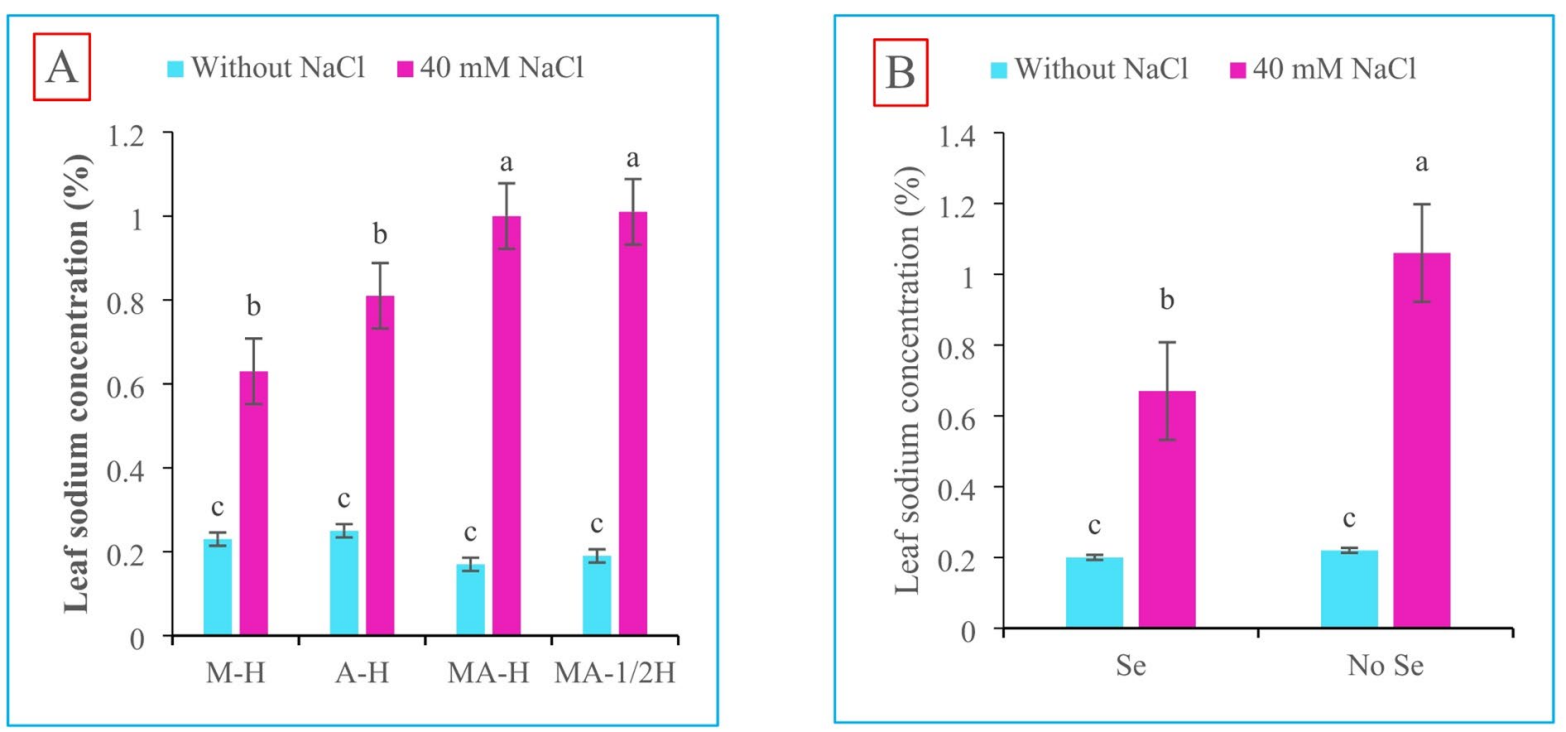
Effect of the two-way interaction of sodium chloride and fertigation regime (**A**), and between sodium chloride and selenium application (**B**), on sodium concentration in strawberry leaves. M‑H: Morning application of Hoagland nutrient solution (daily); A‑H: Afternoon application of Hoagland solution (daily); MA‑H: Morning and afternoon applications of Hoagland solution (every other day); MA‑1/2H: Morning and afternoon applications of half‑strength Hoagland solution (daily). Se: 1 mg L^-1^ selenium; No Se: without selenium. Means (mean ± SD) followed by the same letters are not significantly different according to Duncan’s multiple range test at the 5% significance level (*P* < 0.05).

The interactions between salinity and selenium, and between salinity and fertigation regime, were significant for sodium content (*P* < 0.01). Salinity stress significantly increased leaf sodium concentration compared with the non‑saline treatment, whereas Se application was associated with lower sodium accumulation under salinity. Among the saline treatments, the highest sodium concentration was observed in the salinity MA-1/2H treatment. At the same time, the lowest occurred in the M-H treatment (Fig. 6A). In general, Se-treated groups showed lower sodium concentrations than the Se-free treatments under salinity (Fig. 6B).

### Calcium, iron, and potassium concentrations

The three-way interaction among salinity, selenium, and fertigation regime was significant for calcium, potassium, and iron (*P* < 0.05 for all). Under non-saline conditions, the highest Ca and Fe concentrations were recorded in the MA-H treatment with Se. In contrast, the maximum K concentration occurred in the MA-H treatment without Se. Under salinity stress, the MA-1/2H (0.65%) and M-H (0.63%) treatments with Se showed the highest Ca concentrations. The lowest K concentrations under salinity stress were observed in the MA-H and MA-1/2H treatments without Se, while the highest K (1.19%) and Fe (79 mg kg^-1^) levels were found in the M-H treatment with Se. (Table 3). Among Se-free treatments, M-H also exhibited significantly higher K and Fe than MA-H and MA-1/2H (*P* < 0.05).

**Table 3:** Effect of the three-way interaction of sodium chloride, selenium, and fertigation regime on the concentration of calcium, potassium, and iron in strawberry leaves.

Se (mg L^− 1^)	Fertigation regime	Ca (%)		K (%)		Fe (mg kg^− 1^)	
		Salinity					
		Without NaCl	40 mM NaCl	Without NaCl	40 mM NaCl	Without NaCl	40 mM NaCl
0	M-H	0.81b-f *	0.63e-h	1.62bc *	1.19def	78b *	63b
	A-H	0.72d-h	0.50h	1.51b-e	1.10efg	77b	23d
	MA-H	0.89bcd	0.54gh	2.04a	0.72g	80b	27d
	MA-1/2H	1.04b	0.65d-h	1.85ab	0.74g	80b	28d
1	M-H	0.83b-e	0.78c-g	1.92ab	1.56bcd	84b	79b
	A-H	0.93bc	0.57gh	1.26c-f	1.05fg	81b	56c
	MA-H	1.36a	0.75c-g	1.6bcd	1.19def	115a	51c
	MA-1/2H	0.96bc	0.88bcd	1.54bcd	1.27c-f	89b	53c

* Different letters within each column and row indicate significant differences among means based on Duncan’s test (*p* ≤ 0.05). M‑H: Morning application of Hoagland nutrient solution (daily); A‑H: Afternoon application of Hoagland solution (daily); MA‑H: Morning and afternoon applications of Hoagland solution (every other day); MA‑1/2H: Morning and afternoon applications of half‑strength Hoagland solution (daily).

### Selenium concentration in fruit, shoot, and roots

**Fig. 7:**
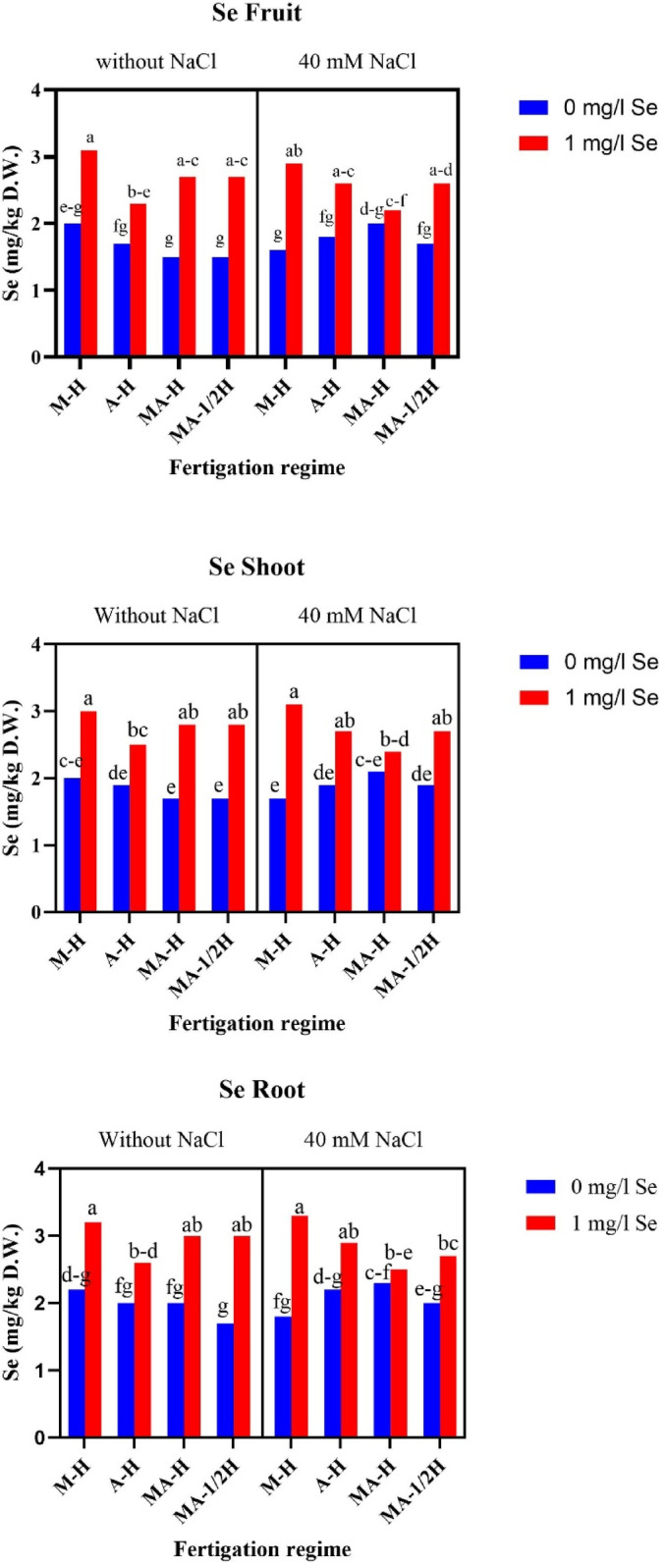
Effect of the three-way interaction of sodium chloride, selenium, and fertigation regime on selenium concentration in strawberry fruit, shoot, and roots. M‑H: Morning application of Hoagland nutrient solution (daily); A‑H: Afternoon application of Hoagland solution (daily); MA‑H: Morning and afternoon applications of Hoagland solution (every other day); MA‑1/2H: Morning and afternoon applications of half‑strength Hoagland solution (daily). Means followed by the same letters are not significantly different according to Duncan’s multiple range test at the 5% significance level (*P* < 0.05).

The three-way interaction among salinity, selenium, and fertigation regime was significant for selenium concentrations in the roots, shoots, and fruits (*P* < 0.05). Supplementation with 1 mg L^-1^ sodium selenate in the nutritional solution consistently increased Se levels in all evaluated tissues across treatments (Fig. 7). Under both saline and non-saline conditions, the M-H treatment with Se resulted in the highest fruit Se concentration, reaching 2.9 and 3 mg kg^-1^ dry weight, respectively. A similar pattern was observed for shoot and root Se concentration. Under non-saline conditions (without NaCl), the MA-1/2H and MA-H treatments with Se showed significantly higher Se concentration in shoots and roots compared to the A-H treatment (Fig. 7).

## Discussion

### Vegetative parameters (leaf area, dry weight of shoots and roots) and yield

The primary innovation of this study lies in revealing how different fertigation regimes interact with selenium supplementation to modulate strawberry responses under salinity stress, rather than merely documenting well-established salinity effects. Our data demonstrate that salinity stress (40 mM NaCl) substantially reduced vegetative parameters, including leaf area and dry weights of both roots and shoots, as well as yield, particularly in treatments without Se (Figs. 2, 3 and 4). These findings correspond with prior reports^5,22,41^. Salinity impairs plant growth primarily through osmotic stress^42,43^, Which inhibits cell elongation and division^44^, and reduces photosynthetic supply to young leaves, ultimately decreasing yield^44,45^. The findings of our study (Figs. 3 and 4) are consistent with these trends.

A critical and novel finding of our study is that under non-saline conditions, the MA-1/2H and MA-H treatments, both containing Se, exhibited the highest vegetative parameters, including leaf area and dry weights of leaves and roots (Figs. 2 and 4). However, regarding yield, the performance was reversed, with the full Hoagland treatment showing a higher yield than the half Hoagland treatment (Fig. 4). This pattern suggests that Se may contribute to improved plant metabolic performance under these conditions, while the fertigation regimes may have influenced water and nutrient availability in the root zone, further supporting growth^46,47^. Se is essential for preventing chlorophyll degradation during stress and for enhancing the capacity of antioxidants in plants^46^. According to the results of Pourebrahimi et al. ^17^ The treatment of Se (1 mg L⁻¹ Na₂SeO₄ applied through the roots) alleviated the negative impacts of salinity on strawberry production by 63% compared to the control treatment.

Since the plants in the present study were developed hydroponically in a substrate containing coir pith and perlite, the twice-daily fertigation regime may have contributed to differences in substrate moisture conditions in the root zone. This condition may have supported more stable water and nutrient availability, promoting optimal conditions for growth. Frequent irrigation can provide a more consistent nutrient supply around the root surface throughout the growth period, preventing the formation of dry zones and supporting more continuous water and nutrient availability^48^. Ismail et al.^30^ demonstrated that dividing water applications between day and night helps maintain substrate moisture, supporting photosynthesis and ultimately increasing yield. This finding aligns with the outcomes of this present research (Fig. 4). Notably, no significant yield reduction was observed when the half-strength Hoagland solution was applied twice daily compared with the full-strength Hoagland solution applied once daily. The same findings have been shown by Pooja et al. ^49^, who found that increasing irrigation frequency enhanced nutrient content in strawberry leaves, shoots, and fruits. Consistent with our observations on fertigation frequency, Hutchinson et al.^50^ recently reported that sensor-based fertigation management in hydroponic strawberries significantly improved yield and water use efficiency compared to conventional timer-based irrigation, with intermediate moisture thresholds (0.225 m³·m⁻³) outperforming both wetter and drier regimes.

According to the findings of this study, under non-saline conditions, fertigation treatment at a half-Hoagland concentration was more effective and economically favorable due to the high cost of chemical nutrients and other inputs. In contrast, under salinity stress, the optimal vegetative and yield parameters were recorded with the daily application of the full Hoagland solution containing Se (M-H treatment). However, a key finding under salinity stress was that applying the nutrient solution twice daily did not enhance strawberry yield in hydroponic conditions. Instead, it caused a significant reduction of vegetative growth and production compared to the single daily application of the complete Hoagland solution with Se (Figs. 2 and 3). We hypothesize that this negative impact may be associated with greater sodium and chloride accumulation in plant tissues (Fig. 6). Frequent application may be associated with differences in salt distribution within the growing substrate, which may adversely affect nutrient balance, plant growth, and fruit development. Although EC was not directly measured in the root zone, this interpretation is consistent with observed ion accumulation patterns (Fig. 6) and previous reports that elevated EC restricts fruit formation and inhibits cell division in fruit tissues^51^.

### Plant pigments (chlorophyll and carotenoids)

Salinity stress reduced chlorophyll concentrations (Table 1), consistent with previous studies^52,53^. Chlorophyll concentration is considered a sensitive indicator of plant health^41^. Increased concentrations of ions of sodium and chloride in the root zone can interfere with the availability and transport of essential nutrients, such as calcium, potassium, and magnesium, thus impairing the formation of chlorophyll and reducing photosynthetic efficiency^54^.

The accumulation of high sodium and chlorine ions in chloroplasts inhibits photosynthesis^55^. Under salt stress, fluorescence parameters are altered, reflecting photosystem II deactivation^55,56^. Our data suggest that Se application was associated with higher concentrations of essential elements (including Mg, Fe, Zn, and Mn) involved in chlorophyll synthesis. As a selenoamino acid, Se also contributes to the formation of chlorophyll precursors^57^. Under salinity stress, the M-H treatment containing Se exhibited the highest levels of chlorophyll and carotenoid indices (Table 1). We propose that this improvement may be partially related to the reduced fertigation frequency, which may have reduced salt accumulation around the roots; this interpretation is supported by the lower Na⁺ concentrations observed in this treatment (Fig. 6). Although root-zone water potential was not directly measured, the maintenance of more favorable water status in this treatment likely supported nutrient acquisition and growth.

Under non-saline conditions, Se-containing treatments exhibited higher pigment concentrations, which may be explained by improved nutrient availability and the efficient absorption of elements essential for chlorophyll synthesis, particularly iron. No significant difference was detected between the two fertigation rounds (full vs. half Hoagland), suggesting that, under non-saline conditions, the presence of Se, rather than nutrient concentration, was the primary factor influencing pigment content.

### Antioxidant activity, anthocyanin content, antioxidant enzymes, and MDA content

Our data indicated that antioxidant activity was higher in Se-containing treatments under both saline and non-saline conditions compared to Se-free treatments (Fig. 5A). Se has two actions that are primarily dependent on its concentration: at low quantities, it promotes the formation of antioxidants, while at high concentrations, it serves as a prooxidant^58,59^. Multiple research investigations indicate that Se supplementation in nutrient solutions promotes antioxidant production in fruits by raising the function or amount of both non-enzymatic and enzymatic antioxidants^60,61^. It may also support photosynthesis performance by boosting photoassimilate generation, potentially facilitating the formation of glycosidic compounds^62^, which may positively influence strawberry anthocyanin content. From an agronomic biofortification perspective, increased anthocyanin content enhances fruit quality and nutritional value, although detailed human health implications are beyond the scope of this study. Salt stress (40 mM NaCl) significantly increased MDA content (Table 2), indicating lipid peroxidation and cell degradation. Water deficit from salinity stress, combined with ionic and osmotic imbalances, promotes excessive ROS accumulation, leading to peroxidation of protein, lipids, and nucleic acids, potentially contributing to cell death^63–67^.

Treatments containing Se significantly enhanced the activity of POD and SOD under salt stress (Table 2). These enzymatic antioxidants protect plants against oxidative damage by neutralizing ROS^68,69^. The molecular reaction to stress initiates biochemical and physiological changes in plants^70^. Se has been reported to influence the expression of antioxidant-related genes, such as Cu-Zn SOD and glutathione peroxidase (GPX), providing additional protection against oxidative damage^46^. In the current study, Se treatments, particularly under salt stress, led to substantial increases in both enzymatic and non-enzymatic antioxidant activities, which may contribute to the protection of the photosynthetic machinery and reduce ROS accumulation. A relationship between Se concentration in leaf tissue and free radical scavenging activity, as indicated in Table 2 This further supports these findings.

The highest antioxidant enzyme activity occurred in the M-H treatment with Se (Fig. 5A). Although root-zone water potential was not directly measured, this treatment may have maintained more favorable root-zone conditions, minimizing water stress and supporting plant water relations. Additionally, the reduced accumulation of harmful solutes in the root zone may have facilitated optimal conditions for plant metabolism and antioxidant enzyme activity. As a result, the plants exhibited better protection of photosynthetic enzymes and more stable membrane integrity under stress conditions.

The decrease in MDA levels in the M-H treatment (Table 2) is consistent with Se’s defensive function in mitigating oxidative damage. Lower MDA levels indicate reduced lipid peroxidation and improved membrane stability, suggesting efficient Se use in mitigating the detrimental effects of salt stress. This treatment’s ability to activate antioxidant systems highlights the potential role of Se in maintaining plant health and enhancing stress tolerance.

### Nutrient concentrations (sodium, potassium, and calcium)

Consistent with our hypothesis that fertigation regimes influence ion distribution patterns under salinity stress, salt stress increased sodium (Na^+^) concentration in leaves, with two-time fertigation treatments showing significantly higher accumulation than other treatments. However, Se application mitigated this effect, a finding that likely reflects improved antioxidant activity and better growth conditions, resulting in reduced Na^+^ concentration (Fig. 6A, B). The lowest accumulation of this ion was observed in the salinity-M-H treatment group (Fig. 6A, B). This reduction may be explained by two factors that are not mutually exclusive: (1) using one-time fertigation instead of two-time, which may have limited excessive ion accumulation, and (2) enhanced photosynthesis and growth through antioxidant action, leading to a dilution effect where the ion concentration per tissue mass decreased.

For potassium (K^+^), our data show that the highest concentration was observed in the MA-H treatment under non-stress conditions, whereas the lowest was observed under salt stress for the same treatment (Table 3). This reduction in K^+^ uptake under stress conditions is consistent with the antagonistic relationship between sodium and potassium, where higher Na^+^ accumulation is often associated with reduced K^+^ concentrations^69^. Furthermore, in Se-containing treatments, K^+^ concentration was higher under one-time fertigation than under two-time fertigation. This observation aligns with previous reports that higher Se concentration in the nutrient solution may reduce K^+^ accumulation^61^. Selenite has been reported to alter membrane permeability to cations, such as Ca²⁺, potentially influencing ion transport processes^60^.

The amount of leaf calcium, which is vital to improving strawberry fruit quality and shelf life, was highest under non-stress conditions in the MA-H treatment with Se. This outcome may reflect both adequate calcium supply in the growth medium and improved transpiration rates, which facilitate Ca²⁺ transport to leaves^71^. Se treatments further enhanced calcium accumulation, possibly by promoting better growth and higher transpiration rates, thereby supporting Ca^2+^ translocation to leaves. Under salt stress, the maximum calcium concentration occurred in the M-H treatment with Se, correlating with favorable growth conditions (root and shoot dry weights; Table 3). Conversely, the lowest calcium level under stress was observed in the A-H treatment, which may be attributable to suboptimal growth and reduced nutrient uptake associated with the single fertigation event. Although root-zone ion dynamics were not directly measured beyond tissue concentrations, these patterns are consistent with our observed growth and yield responses (Figs. 3 and 4).

### Selenium concentration and biofortification

Under salinity and non-salinity situations, the concentration of Se in fruits, leaves, and roots increased when sodium selenate (Na₂SeO₄) was added to the nutritional solution (Fig. 7). The Se content followed the order: root > shoot > fruit. Among all treatments, the M-H treatment with Se exhibited the highest Se concentration across all plant tissues. Under non-stress conditions, one-time fertigation resulted in higher Se accumulation in fruits than two-time fertigation. This may reflect a concentration effect due to reduced plant growth rather than increased uptake. Conversely, under salinity stress, fruit Se content was higher, possibly due to improved root health and more favorable nutrient acquisition conditions.

Both excessively rapid and significantly reduced plant growth can affect the accumulation of minerals, including Se, in plant tissues. Applying selenite to plants allows plant tissues to absorb and metabolize Se compounds^72^. These compounds, like selenomethionine (SeMet) and selenomethyl-cysteine (MeSeCys), are transported through the symplastic pathway to leaves, fruits, and other tissues^73^. Se can also be transported as selenate, the oxidized form of selenite, for redistribution from leaves to fruits^74^.

Consistent with previous observations^61^ Se concentration in soil-grown plants was lower than in perlite- cultivated plants. This is likely due to the strong adsorption of selenite by metal oxides in soil, which reduces Se availability to plants^75^.

Se levels in control treatments (no Se supplementation) were calculated from the baseline Se levels in the growing substrate and irrigation water. Our study demonstrated that strawberry plants can absorb and store substantial quantities of Se in their organs without inducing toxicity or impairing vegetative and reproductive parameters. Therefore, supplementing strawberry nutrient solutions with Se serves two purposes: (1) mitigating negative impacts of salt stress and promoting plant development under these conditions, and (2) enhancing biofortification, a strategy to address Se deficiencies. Se biofortification has been successfully demonstrated in numerous crops, including radishes^76^, potatoes^27^, and strawberries^5,77^.

From an agronomic perspective, the measured Se concentrations (2.9–3.1 mg kg^− 1^ D.W. in fruits from the M-H treatment) indicate successful biofortification. These values are comparable to or higher than those reported in other strawberry biofortification^5,77^. The Se concentrations achieved in this study fall within ranges considered safe for plant growth and are consistent with biofortification targets; however, detailed dietary recommendations require consideration of total Se intake from multiple food sources^78^ and are beyond the scope of this agronomic study.

### Limitations and future research directions

Although the present study provides valuable insights into the roles of selenium and fertigation regime in modulating strawberry responses to salinity stress (40 mM NaCl), several limitations should be considered to properly contextualize the findings. First, the experiment was conducted on cultivar ‘Paros’. In contrast, genetic differences among cultivars may lead to substantial variation in salinity tolerance, selenium uptake, and utilization, thereby limiting the generalizability of the results. Second, we tested only the selenium concentration (1 mg L⁻¹), which does not allow evaluation of dose–response relationships or identification of threshold or potential toxicity levels. Third, the study employed a fixed set of fertigation schedules. Although these schemes provided valuable information, they cannot fully capture the diversity of diurnal physiological rhythms, evapotranspiration dynamics, and circadian‑regulated nutrient transport and acquisition processes, all of which may influence strawberry responses to salinity, selenium, and fertigation regimes. Fourth, elemental concentrations ascertained by SEM-EDX should be interpreted carefully because this method provides semi-quantitative estimates and is more suited for treatment comparison than for precise measurement of tissue mineral concentrations. Furthermore, the experiment was conducted under controlled greenhouse conditions using a specific substrate system; therefore, the results may not reflect the complexity of open‑field environments where soil heterogeneity, climatic fluctuations, and biotic interactions play significant roles. Accordingly, future research should investigate a broader range of selenium concentrations, compare multiple cultivars with different levels of salinity tolerance, and explore more diverse fertigation strategies across the diurnal cycle. Field‑based and long‑term experiments are also necessary to determine the stability and agronomic relevance of selenium‑induced responses under real production conditions. Advancing research in these directions will contribute to a more comprehensive understanding of the combined effects of selenium and fertigation regimes under salinity stress in strawberry cultivation and support more robust and practical recommendations for growers.

## Conclusion

This study demonstrated that salt stress greatly decreased vegetative growth and fruit yields in strawberries. However, optimizing both selenium supply and fertigation regimes effectively mitigated these adverse effects under the conditions tested. Under non-saline conditions, the two-time application of half-strength Hoagland solution supplemented with selenium was associated with improved nutrient status, improved photosynthetic performance, and strengthened antioxidant capacity. Under saline conditions, a single morning application of full-strength Hoagland solution with selenium was more effective than the other treatments evaluated in this study, improving nutrient balance while boosting both enzymatic and non-enzymatic antioxidant systems. These improvements supported better vegetative development, higher yield, and enhanced fruit quality within the framework of our experimental system. Moreover, all selenium-enriched treatments resulted in meaningful selenium biofortification in the fruits, suggesting that under similar conditions, appropriate fertigation regimes can simultaneously enhance stress tolerance and improve nutritional value. Future research should investigate whether these regime-dependent responses are consistent across different cultivars, growing systems, and environmental conditions before broad recommendations can be made. Understanding the physiological mechanisms driving diurnal sensitivity to nutrient and selenium uptake could further refine fertigation strategies. Long-term field studies and dose–response analyses will also help determine optimal selenium inputs that maximize fruit quality and biofortification without compromising plant performance under commercial production conditions.

## Data Availability

The datasets generated and/or analysed during the current study are available from the corresponding author on reasonable request.
